# Child-led enquiry in primary science

**DOI:** 10.1080/03004279.2013.822013

**Published:** 2013-08-01

**Authors:** Lynda Dunlop, Kirsty Compton, Linda Clarke, Valerie McKelvey-Martin

**Affiliations:** ^a^ Faculty of Education, Liverpool Hope University, Liverpool, UK; ^b^ School of Biomedical Sciences, University of Ulster, Coleraine, UK; ^c^ School of Education, University of Ulster, Coleraine, UK; ^d^ School of Pharmacy and Pharmaceutical Science, University of Ulster, Coleraine, UK

**Keywords:** philosophy for children, science education, community of enquiry

## Abstract

This research describes and evaluates the application of a child-led approach to scientific enquiry (the Community of Scientific Enquiry, CoSE) to children aged 8–11 (Key Stage 2) in Northern Ireland. Primary teachers were introduced to CoSE at a workshop and asked to evaluate its implementation with their class. Results from children (*n* = 364) and teachers (*n* = 19) found that CoSE engaged children with their science learning, and also developed confidence and oracy. However, teachers require more experience developing facilitation skills and in fitting science into a thematic teaching unit.

## Introduction

The study began in 2008 against a backdrop of a period of major curriculum reform in Northern Ireland. Although the purpose of the revised curriculum was to build on an understanding of how children learn and develop (Council for the Curriculum, Examinations and Assessment (CCEA) 2005), the place of science education in this revised curriculum presented some challenges to science educators. Science was removed as a stand-alone subject in the primary curriculum for all year groups from 2009 (CCEA 2012) and incorporated (with history, geography and technology) into the ‘learning area’ *The World Around Us* (WAU). This is taught through cross-curricular thematic units, with substantial freedom for teachers to decide if and how they teach science.

However, the selective education system has the potential to stifle this freedom. During the final year of primary school, children who wish to attend a selective secondary school sit a transfer test. Although the state transfer exam was abolished in 2010, grammar schools continue to control admission by means of examination. This is currently administered by two private organisations on behalf of the selective schools. These examination papers contain questions on English and mathematics only, whereas the previous (state administered) transfer exam included science. Although primary teachers are recommended not to prepare children for unregulated transfer tests (Department of Education 2010), anecdotal evidence suggests that they do. It is possible therefore, that the primary curriculum at Key Stage 2 is distorted in favour of English and mathematics to the detriment of other subjects. Indeed, a recent survey of primary teachers in Northern Ireland found that less science is being taught now that science does not exist as a subject in its own right, and in some schools science is not being taught at all (Green, Dunlop, and McKelvey-Martin 2011).

A further problem facing science education in Northern Ireland is that of engagement: it has been found that science education fails to engage young people (Millar et al. 2006;  Department for Education and Department for Employment and Learning  2009). Little research has concentrated on the evaluation of strategies to address this problem in school science (Osborne, Simon, and Collins 2003).

The aim of this research was to develop a strategy for the teaching and learning of science that is sensitive to the aims of science education, the needs of learners and to findings from science education research, which has often been criticised for its limited influence on practice (Millar et al. 2006). The *Community of Scientific Enquiry* (CoSE) strategy developed in this research responds to curriculum changes by focusing on enquiry skills in a scientific context. Resources such as Science and Technology in Society (SATIS) exist to support the teaching of how science works between the ages of 8 and 19 (Association for Science Education 2008). SATIS is concerned with making science relevant: to the future citizen, the future worker,  students' own lives and the wider world (Holman 1986). The SATIS resources are units of study in the form of worksheets that introduce a range of teaching and learning activities, and discussion is just one strategy among group work, role play, problem-solving, reading and practical work, there being no common structure to the SATIS materials. The guide for teachers is comprehensive, consisting of guidance on starting and running a good discussion. A survey-based evaluation of the use of SATIS by teachers at post-primary stages of education (Watts and McGrath 1998) found that SATIS materials were most effectively used in the context of data analysis. The SATIS activities are useful for making children consider the issues at play, but the discussion is highly directed and leaves little room for their own opinions on the matter to be explored. SATIS has recently been updated and is without doubt a valuable resource for integrating how science works into school science. However, it is not a single strategy and discussion is sometimes a minor part of the activity, although selected SATIS resources could be incorporated into a CoSE discussion.

The research was timely in that the reform of the curriculum presented the socio-cultural context in which to evaluate the strategy. CoSE is a development of *Philosophy for Children* (P4C) (Lipman 2003; Lewis and Chandley 2012) and is concerned with enabling children to participate in dialogue concerning one or more of the following: the *content* of science (science concepts), the *nature* of science (philosophy of science) and the *consequences* of science (social, ethical and technological).

This paper presents the origins of CoSE as a development of P4C, illustrates what CoSE looks like in practice, and reports on the evaluation survey of CoSE by children and their teachers.

## From P4C to communities of scientific enquiry

The CoSE strategy has its origins in P4C, advocated in the UK by the Society for the Advancement of Philosophy and Reflection in Education (SAPERE). Philosophical dialogue is distinguished from discussion (McCall 2009) in that it contains more than one philosophical idea, and it involves contrasting philosophical ideas which are supported by argument and a movement or development of ideas and arguments. P4C as advocated by SAPERE has a common structure consisting of the following sequence of steps (Williams 2012):

- *Preparation for the enquiry*: arranging seating, sharing ground rules and warm-up activities to support co-operation, speaking, listening and thinking.
- *Stimulus*: presented to the class in order to engage them and to provoke them to raise interesting ideas and questions.
- *Thinking time*: to reflect individually and in pairs, about the issues raised.
- *Question making*: in which the class is split into small groups to create open-ended questions.
- *Question airing*: explaining and clarifying questions to the whole class and linking and evaluating these questions.
- *Question choosing*: voting for a question to begin the dialogue or creation of an agenda.
- First thoughts in the dialogue are invited by the authors of the question selected.
- Building dialogue brings in the ideas of others in the class. The teacher/facilitator or children themselves can invite, for example, comments, reasons, examples, agreement and disagreement, and other perspectives.
- Last thoughts in the dialogue allow everyone to share their final idea, although participants may pass if they wish.
- *Review*: reflecting on the process of enquiry and identifying successes and areas for development.

Often advanced as a strategy for promoting thinking skills rather than philosophy, there exists research evidence that the community of enquiry approach is successful for promoting learning, reasoning and self-esteem in young people (Trickey and Topping 2004; Thwaites 2005). Furthermore, there is an acceptance that children's use of enquiry skills is related to the development of their conceptual understanding (Harlen 2009). The pedagogical strategy used during the philosophical enquiry is the community of enquiry.

## Community of enquiry

People

… live in a community in virtue of the things which they have in common; and in communication is the way in which they come to possess things (aims, beliefs, aspirations, knowledge] in common. (Dewey 2007, 4)

A community can be understood to be a unit of a social organisation consisting of a group of people who share an identity and sense of pursuing common goals (Poplin 1979) as well as sense of belonging and need to participate (Wenger 1998). Thus, every school class is a community. As Dewey highlights, the community is developed through communication. Lipman (2003) characterises the particular nature of a community of enquiry: that it is self critical and aims to produce a judgement (albeit tentative) through structured dialogue which is characterised by care and collaboration as well as critical and creative thinking. The type of behaviour seen is children listening to one another, building on their peers' ideas and challenging unjustified beliefs by asking for reasons. In Lipman's community of enquiry, the dialogue goes where the enquiry leads and is free from disciplinary boundaries. Fisher (2005) stresses that the purpose of a community of enquiry is to join together to address a question of common concern, and through the exchange of different points of view, to reach a better understanding.

The community of enquiry approach offers children the opportunity to decide what they think is important to discuss based on the stimulus provided by the teacher and eschews the direct teaching of children. It prioritises their thinking. It is recognised that a correct answer may not be found, nor may the community reach a consensus view. What is of value is the process, and the new knowledge and understanding that children gain from participating in the community of enquiry. The central idea of the community of enquiry is to provide a group learning environment in which children co-operate to test, share and improve on their thinking together (Splitter and Sharp 1995). These enquiry skills in a scientific setting include hypothesising, observing, measuring and recording, predicting, raising questions, communicating verbally and non-verbally and self-criticism (Harlen 1977). The CoSE and development of these and other skills is brought about by a teacher who facilitates the enquiry.

### Facilitation in a community of enquiry

The role of facilitator requires, perhaps more than anything, the disposition described by Paulo Freire:

The educator is not he who knows, but he who knows how little he knows, and because of this seeks to know more, together with the educatee, who in turn knows that starting from his little knowledge he can come to know more. (Freire 1975, in  United Nations Educational, Scientific and Cultural Organisation 1997, 161)

That is, they must be intellectually honest when they do not know an answer and be prepared to seek answers, or enquire, with others. P4C practitioners have described the facilitator's role in a variety of ways. In many ways, this is a similar role to the characteristics of a good teacher. The facilitator mediates (Lipman 2003), acts as a discourse guide (Mercer 2008) and co-enquirer (Gardner 1996). These different conceptions have in common that they highlight the need for the teacher/facilitator to create the environment for the enquiry (Palsson, Sigurdardottir, and Nelson 1999), to model skillful thinking (Sprod 2001), to focus the discussion, and to encourage deep consideration of the topic through the use of procedural questions (Gardner 1996). Although not referring to P4C, Bohm (1996) proposes that the ultimate role of the facilitator is to work themselves out of a job: the members of the community should end up facilitating their own enquiry. Facilitating in a community of enquiry has also been likened to Socratic teaching (Bramhill 2002) in that it is non-autocratic, and involves listening to, and drawing out, the children's views. There is a mutual responsibility for the development of dialogue, and the teacher helps the child to express and reflect upon their viewpoint by asking questions and identifying inconsistencies in argument. In the context of their review of how teachers can enable a good-quality discussion in science, Levinson, Hand, and Amos (2012) also highlight the role of the teacher in promoting critical enquiry, developing children's reasoning skills and as a knowledge resource.

There is considerable diversity of practice when it comes to facilitation. Some prefer to intervene more, and others prefer the community to come to manage itself. According to Burden and Williams (1998), the facilitator must be *pedagogically strong but philosophically neutral.* On the other hand, Fisher (2005) identifies the teacher in three different roles in a community of enquiry: as participant, facilitator and philosophical expert. There is considerable diversity in practice in terms of how much the teacher contributes to the dialogue in an ‘expert’ capacity and also in the extent to which participants are encouraged to contribute to the dialogue, not only through listening.

In adopting the role as facilitator, it must be remembered that learners prefer their teacher to indicate differences in values but also to express the values that they find important, i.e. to be intellectually honest, enabling them to filter what teachers say and do in awareness of the teacher's perspective (Halstead and Pike 2006). There are some risks and tensions associated with the role of teacher as facilitator. In the first instance, the focus on the democratic process hands over control to the children, so the questions cannot be guaranteed to fit in with specific curriculum objectives, yet stimuli are selected in anticipation of particular responses. Additionally, there is the question of how to deal with enquiries that go round in circles, educators wanting to start with definitions before the ‘proper’ discussion can begin, and dealing with taboo and unexpected topics (Murris 2000).

## P4C in science education

There are a number of proposed benefits of P4C to science education cited by the originators of the approach: that P4C embraces the spirit of science by encouraging a critical temper of mind and the questioning of facts (Lipman, Sharp, and Oscanyan 1980). Some elements common to philosophical and scientific enquiry include generating questions, suggesting hypotheses, giving reasons and examples, making distinctions and connections, analysing implications, devising and using criteria and forming arguments that are consistent.

A generic account of the application of P4C to science can be found in Lewis and Chandley (2012), which describes how philosophical enquiry can be used in science to support children's understanding of the four connected themes of nature of science, scientific discovery, ethics in science and of scientific concepts. Two UK-based research studies of P4C exist, highlighting its potential for engagement with science and scientific reasoning skills. Bartley's (2004) study used children's self-assessment questionnaires to evaluate P4C as the second part of a two lesson sequence on genetically modified (GM) crops (the first lesson used print media and a class discussion on GM crops). Bartley found that learners better understood how GM crops were produced (70%) and that they could discuss the benefits and concerns with others (65%). However, it is difficult to disentangle the impact of P4C from the media-based discussion, particularly because the questions selected for the P4C enquiry were *can human DNA be changed?* and *what is life?* were not directly connected to GM crops. Sprod's (1998) study of P4C in science education in England involved the comparison of scientific reasoning skills of a class of year 7 (first year of secondary school) children with a control class who did not experience P4C. Sprod found significant improvements in scientific reasoning in the experimental group compared with the control group.

Arguments in favour of the use of P4C in science are that it helps children to create meaning by allowing them to clarify concepts and by enabling them to link scientific ideas with other ideas,  promotes understanding of the nature of knowledge (including science knowledge), permits them to explore socio-ethical issues and  gives them access to philosophy as a discipline (Sprod 2001).

However, there are a number of limitations to the application of P4C in the science classroom. These relate to the *content* and *process* of philosophical enquiry in contrast to scientific enquiry.

In terms of content, philosophical concepts have been described as common, central and contestable (Splitter and Sharp 1995); i.e. philosophical concepts such as truth, justice and knowledge are familiar to most people, at least to some extent, and they are central to what it means to be human. Scientific concepts on the other hand are often unfamiliar, abstract and removed from children's lived experience, and young people need help to apply scientific knowledge to a new concept. While some scientific concepts such as that of biological species are contested, many have precise definitions and are related to other scientific concepts in rigidly defined ways. For example, weight is the force that acts on a mass due to the action of gravity. Scientific conceptual differences can ultimately, if not immediately, be resolved by recourse to experimental evidence. Philosophical concepts on the other hand cannot readily be resolved: there is no authority or foundational principle for philosophical concepts (Williams 2012).

Philosophical concepts are also more open for discussion. That is not to say that scientific concepts cannot be the subject of productive dialogue in the science classroom, but that a differentiation between more contestable and less contestable concepts is required by the facilitator. P4C focuses on the exploration of philosophical concepts and works towards the recognition of complexity and an appreciation of where it is not possible to reach a consensus (Williams 2012). The researcher's experience of P4C was that children tended to select non-scientific philosophical questions, by which is meant philosophical questions that are detached from science. Examples of such questions are *are humans free?* and *is there a God?* While children might enjoy their lessons and learn more, what they are enjoying and learning about is not science. It is philosophy.

Enquiry in philosophy and science education has a common basis. It is:

The process of students asking relevant questions about issues to which they do not posesss predetermined answers. (Sutman 2000, 8)

This definition highlights two important aspects of enquiry in education. It is based on questions, and the answers to these are unknown *to children*. In philosophical enquiry, it may be the case that the answers are unknown *full stop*. This is also possible in scientific enquiry, but it is more likely that many of the answers are known tentatively by the scientific community. Philosophical enquiry does not rely on conceptual knowledge to as great an extent as scientific enquiry, and when used in education, there is often no definitive answer to reach. However, there often is an accepted answer to children's enquiry questions, and the challenge is to encourage children to reach this themselves through, for example:

Observation, imagination and reasoning about the phenomena under study … the use of tools and procedures … that allow students to extend their everyday experiences of the world and help them organise data in ways that provide new insights into phenomena. (Bransford and Donovan 2005, 405)

P4C is beneficial in science teaching when it addresses philosophical issues in relation to science, but is limited in its approach to scientific concepts and therefore to meeting the needs of science education. It was therefore necessary to design a strategy that harnessed the strengths of P4C but that also addressed the need for teaching and learning about scientific concepts.

If good thinking is to become the prime objective of the classroom, is it to be along the lines of scientific enquiry or philosophical enquiry? Philosophy and science are independent ventures, in no way reducible to one another. (Lipman 2003, 36)

In a science classroom, the answer to Dewey's question must be that *scientific* enquiry is to become the prime objective of the classroom. For that reason, CoSE was developed from P4C.

## Community of Scientific Enquiry (CoSE)

CoSE is a way of promoting teaching and learning science through enquiry, driven by children's own questions which are explored through dialogue. The CoSE strategy is concerned with enabling children to participate in dialogue concerning one or all of the following: the *content* of science (science concepts), the *nature* of science (philosophy of science), scientific *discoveries* and the *consequences* of science (social, ethical and technological). The CoSE strategy consists of a sequence of steps similar but not identical in appearance to P4C, and quite different in emphasis. This common structure is applied to the design of supporting resources:

- Information
- Starter
- Stimulus
- Question creation
- Question selection
- Enquiry
- Evaluation
- Consolidation

A CoSE class begins with the children and teacher preparing for the enquiry. Children may be given information in advance of the enquiry, or asked to revise or research the scientific content under question. This may be set during the previous lesson or as homework. Alternatively, the group can approach the enquiry without this contextualisation, particularly if the teacher is using CoSE to ascertain children's existing ideas about a topic. The teacher prepares the environment by making sure that all members of the group can see and communicate directly with each other and the facilitator, and are able to participate in the starter and stimulus activities. This also has the effect of marking out a space and time for a different type of learning activity. Evidence exists that physical barriers limit participation in lessons (Weinstein 1979). One way of removing the physical barriers in a lab is to sit in a circle or horseshoe shape with no tables or benches in front of the children. Where not possible in a classroom, an open enquiry space such as a library or drama studio may be used.

The CoSE class starts with an activity to revise key scientific concepts and to warm up for discussion. A science-based stimulus such as an experiment or demonstration, music or comic strip is then shared with the group. In response to the stimulus, each child generates a question that they are interested in exploring. These are then shared with other children in small groups, who select one question to put forward to the whole class. The class votes on the question they most want to discuss. Varied voting strategies are used. Depending on the question selected, the teacher might postpone the enquiry to send children away to do research. For instance, if the question *if we clone humans will evolution stop?* is selected and the class has not yet learnt the mechanism of evolution, the enquiry might benefit by giving children time to go away, investigate, think and talk about evolution by natural selection before trying to apply it to reproductive cloning.

The dialogic enquiry follows, facilitated by the teacher. The learning that has taken place is reviewed at the end and children are then asked to complete an exercise that consolidates their understanding of key scientific concepts in the context of what was learnt during the enquiry.

Whilst both community of enquiry and CoSE are pedagogies for promoting learning, the ‘flavour’ of a community of enquiry is philosophical: the emphasis is on exploring philosophical questions like the aforementioned *what is life?* CoSE is less philosophically deep, but more scientifically rich than P4C. It is focused on scientific questions. At times, these may draw in philosophical issues relating to the nature of science or science ethics, as is the case when exploring ethical issues in science. However, the differences between scientific and other ways of knowing are highlighted, and there is a focus on what scientific evidence exists, and how this is weighed against other factors present in a given scenario. At the core of CoSE is the concentration on children's scientific ideas and how these relate to mainstream, contemporary scientific ideas. In a philosophical community of enquiry there can be many correct answers, some more valid than others; whereas in CoSE there may be a ‘correct’ answer in response to the question selected for enquiry, and in this case the enquiry focuses on competing ideas from children, the evidence for different ideas, and what would have to happen in order to falsify each.

Frequently, however, questions in CoSE will be conceptual questions, relating to key ideas in the sciences. Scientific enquiry is evidence-based and concerned with how these empirical facts are interpreted within the currently accepted framework of scientific understanding (Lipman, Sharp, and Oscanyan 1980).

Enquiry in science is often taken to mean practical enquiry, but enquiry through dialogue has much to offer the science teacher and learner. The dialogic enquiry process allows children to make their ideas and understanding explicit; it allows them to express ideas in their own words, to bring knowledge from other areas of the curriculum to inform scientific decision-making; it highlights uncertainties in science and provides a space to explore the social and ethical issues inherent in science. The key element of the CoSE strategy is the creation of children's own questions in response to a stimulus and the search for answers to these questions through dialogic enquiry. Although talk is central to CoSE, this does not preclude the community from advancing their investigation through other methods such as investigation based on their own ideas as outlined in the SPACE project (Black and Harlen 1993) or along the lines of a structured investigation as outlined by Goldsworthy and Feasey (1998).

## What does CoSE look like?

The following section describes what CoSE might look like to an observer, the teacher of a class, and to the children in the class. Although idealised, and highlighting aspects of CoSE rather than the full detail of the primary science classroom, the following vignettes were created from a synthesis of the researcher's experiences of, and reflections on, facilitating CoSE classes and demonstrate *some* of the distinctive features of CoSE as an approach to science education that may be evident following a sequence of classes using this approach.

### to an observer …

The classroom is arranged with seats in a circle. There is dialogue relating to a question on the floor in the centre of the room, but the dialogue moves around the room. It is not immediately obvious who the teacher is, but for the occasional prompting for ideas, reasons and examples when the children do not get there first, or when a child seeks confirmation of some factual information. Children are using scientific language and at times hesitate, check they have used vocabulary correctly, and carry on to develop their point. At times there are furrowed brows, perplexed faces and silence, at other times there is laughter, animation and activity as children pitch in to add to what someone else has said, disagree or put forward their idea. Disagreement is with reference to ideas, not people. Children are listening to each other and expressing themselves freely. They are concentrating on the subject matter of science, making connections between abstract ideas and their own lives, referencing previous lessons, life experiences and personal values. The question might not have been answered at the end of the enquiry, but scientific ideas have been explored, children's ideas relating to those ideas have been aired and evaluated, and there might be a suggestion on the table as to *how* the question could be answered (or indeed, *if* it can be answered using science). As the children leave the classroom, they are absorbed in talking about ideas generated during the enquiry with each other.

### to the teacher …

For once the attention of my class is not solely on me. I still can relax though. They are meant to be learning about nutrition, and we have tried some edible insects, but what happens if their enquiry question is off topic? That is time we can waste. We look at the edible insects advertisement stimulus and there is silence as children make up their questions, then disagreement, discussion and ultimately compromise as small groups choose one to put forward to the vote. The class chooses a question that doesn't relate directly to nutrition: they want to know what would happen to other animals if we farmed insects instead of sheep and cows. I give them time to think and discuss in small groups before asking the group that generated the question to get us started with some ideas. The discussion is slow to get started, and needs me to tease out ideas from different children, but before long children are applying what they know about food chains and webs to this hypothetical situation. I pick up on a few misconceptions. Some are corrected by their peers with a better understanding than them, others are not directly relevant so I make a note to bring these up in a later lesson, and for yet others I have to do a bit of probing with questions to encourage children to think harder about their ideas. At times the dialogue flags and I have to encourage them to think of the question from a different perspective; at other times the talk has gone off at a tangent and I have to refocus on the question. At one stage, the class reaches a consensus and seems quite content until I ask them what would make them change their mind, which sets them off again thinking about what evidence would be needed. The children raise many questions during the enquiry; some of which I can answer simply without interrupting the flow of enquiry or turning it into a question-and-answer session, others need a more detailed explanation that I will save for a later date. At least that is some lesson planning done, and I have a much better idea about what my class understands well and what needs further work, and I have some interesting ideas about what they would do given free reign in a science lab. I know them better. They haven't answered the question, but yet are wiser for having tried.

### to a child …

There are no books. That means no writing. We have to sit in the circle and have the chance to try an insect. I do not like the look of them so I don't have one. We read an advertisement about eating insects. I had never really thought about how it was normal to eat insects in other places, or about how it was better for the environment. We have to look at the advertisement and make questions up for our discussion. I wanted to know why we were brought up to not eat insects if it was such a good thing. It wasn't chosen so I wasn't happy at first but the question we did choose was about what would happen if we started farming insects instead of cows and sheep so it was interesting to think about. I didn't want to talk at first, but the teacher asked me if I agreed with what someone else had said so I had to speak up and give a reason for what I said. Someone else agreed with me and gave an example too so we were able to argue with what some other people were saying. It was strange because I was agreeing with people I don't normally speak to. We talked about new science, if you could ever be proved right, how the government makes laws, how we know if something is safe, and what different animals eat. We even came up with a way to find out if insects were fatty! We didn't have to listen much to the teacher today, but we still learnt a lot from other people. It wasn't what I had expected to learn in science today but it was good fun and I got to hear what other people in my class thought. I still want to know why we are brought up to think insects are disgusting though!

The character of each classroom community is different, as are the questions of interest to the community. The characteristics of a CoSE lesson are therefore different for each group. However, the emphasis is always on the process of enquiry, and what science contributes to knowledge. Although specific content cannot be ‘taught’ through a CoSE, the teacher must ensure that children learn.

### The role of the teacher in a CoSE

The fundamental role of the teacher in CoSE as in any aspect of education, is to support children in their learning. They must be alert to the teachable moment and critical incidents, and must inspire children with love of learning and enquiry (von Glasersfeld 2002).

During the starter, the teacher's role is to create the social environment for learning. This is achieved through a game that focuses on scientific content and encourages behaviour for learning such as listening to one another, participation and correcting and challenging each other respectfully.

When presenting the stimulus the teacher explains the link between key scientific concepts and the topic under consideration. For example, the link between food chains and nutrition and edible insects. During the presentation of the stimulus, children are encouraged to ask questions to clarify their understanding of the topic.

In P4C, questions created by children are completely open, whereas in CoSE children are asked to create a question that relates to the target scientific concept. Facilitating the dialogue in a CoSE involves encouraging the following among children: clarification of question, definition of key terms, identification of evidence/knowledge required, constructing an argument including reasons, examples and counterexamples, thinking of alternative views, ideas and solutions; and encouraging children to participate in different ways such as contributing to the dialogue (for more reticent children), and listening to peers (for children who tend to dominate during discussion). It also involves highlighting scientific concepts and aspects of how science works relevant to the dialogue. This is achieved primarily through questioning. The types of question that a teacher might use frequently when facilitating a CoSE class include: *how do scientists know that?, what is the evidence for that?, what would prove that wrong?, what discovery would change scientists' minds?, what other hypotheses are there? and how could that idea be tested?*

During the enquiry, it is important to give the community the opportunity to identify and correct children's misconceptions, and to begin to regulate its own behaviour. This is an important step towards children taking responsibility for their own learning. That is not to say that misconceptions are not dealt with. In the ideal scenario, children correct one another using language and ideas with which they are familiar. Alternatively, the teacher can provoke this by focusing the dialogue on a specific misconception and prompting thinking based on questions that challenge erroneous ideas. If the misconception is not directly relevant to the discussion, it might be better noted down and returned to in a later lesson.

Some of the challenges that have to be addressed by the facilitator include the avoidance of ‘cosy’ dialogue (Bohm 1996) in which consensus is reached too readily and without due challenge of ideas; managing the diversity of opinions so that children do not become overwhelmed or frustrated with the number of ideas under consideration; encouraging participation in different ways so that quieter children gradually contribute more, and more assertive children dominate less; and, controlling the pace of the dialogue so that ideas are explained carefully and accurately but not over-laboured.

## Evaluation of CoSE

To evaluate CoSE with children at Key Stage 2, teachers were introduced to the approach at a workshop where they participated in enquiries and were provided with sets of resources to enable them to run 8 CoSE sessions with their class. At the end of the series of CoSE classes, children were asked to complete a questionnaire. Findings from the evaluation with children (*n* = 364) were validated by asking their teachers to evaluate the approach (*n* = 19).

## Results

### Engagement of children at Key Stage 2

The evaluation questionnaire administered to children contained the following items:

- Did you talk about the topics after class with others? (item 1).
- I enjoyed the CoSE classes (item 2).
- The CoSE classes were interesting (item 3).
- The CoSE classes were boring (item 4).

A coefficient of reliability was calculated using items 1–3 and item 4 in reverse (Cronbach's *α* = 0.695) to estimate the reliability of the items as a measure of engagement. Although the value of Cronbach's *α* increases to 0.762 if item 1 is deleted indicating a better measure of engagement, the item was retained because action beyond the classroom is a demonstration of an action as a result of deeper engagement with the subject.

#### Taking learning beyond the classroom

Eighty per cent children agreed or strongly agreed that they knew more about science after the CoSE classes. The majority (63%) said that they talked about the topics after class with parents, other family and friends. The following are indicative of children's comments on returned questionnaires:

I loved all of the classes. I would try them at home because they made me think about science. Now I am looking forward to science next year in high school.

We found out stuff and you could show your parents at home.

#### Enjoyment

**Figure 1.**
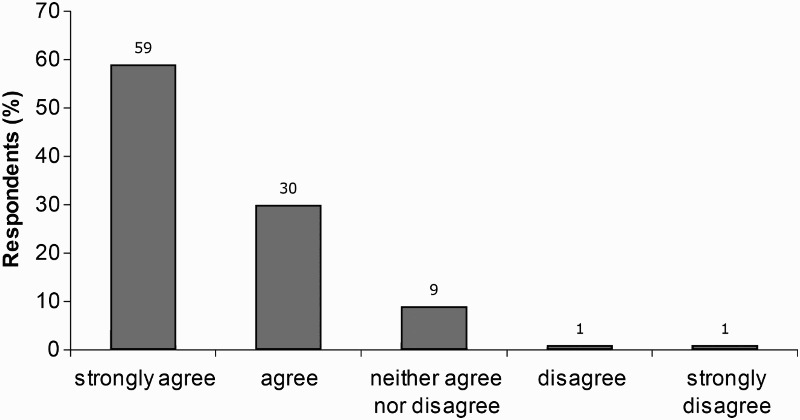
Children's response to item 2 ‘I enjoyed the CoSE classes’.

Most children (89%) agreed or strongly agreed that they enjoyed the sessions (Figure 1), exemplified by the following:

Science is really exciting and fun. I wish I could do loads more!

I very much enjoyed [CoSE] and think it was a very good idea as the normal lessons in primary school don't include science.

I really enjoyed the lessons and I had fun because you were allowed to talk and discuss your answers and opinions.

#### Interest

**Figure 2.**
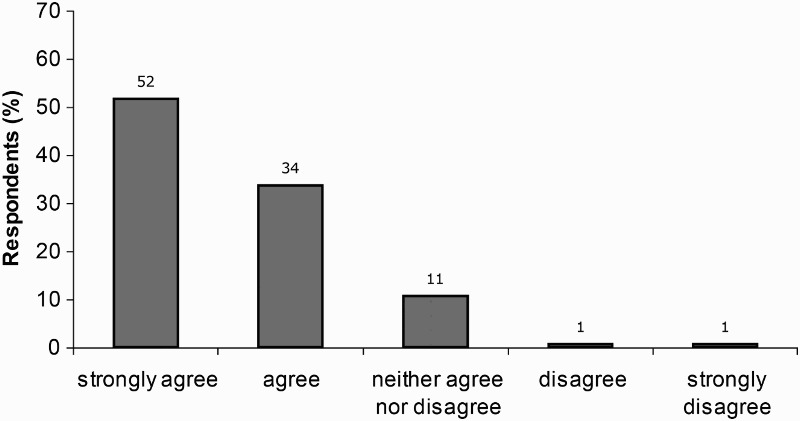
Children's response to item 3 ‘I found the CoSE classes interesting’.

**Figure 3.**
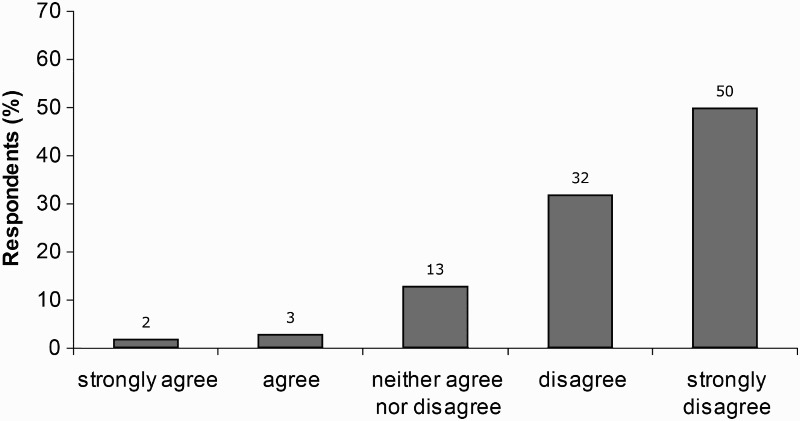
Children's response to item 4 ‘I found the CoSE classes boring’.

Most children found the CoSE sessions interesting (Figure 2): 86% agreed or strongly agreed with the statement ‘I found the CoSE classes interesting’; while only 5% agreed or strongly agreed with the statement ‘I found the CoSE classes boring’ (Figure 3).

All of the classes were very interesting and brilliant.

Doing these experiments has made me more interested in science. Science is now one of my favourite subjects. I've enjoyed working in groups so we can share our ideas.

Qualitative data on the best and worst aspects of CoSE as identified by children in all participating schools were coded and are presented in Tables 1 and 2, respectively. This indicates *why* so many were engaged by their CoSE classes.

**Table 1.** Best aspects of CoSE for children at Key Stage 2.

*Engagement*	
Emotional engagement	Children emotionally engaged with CoSE. They described it as interesting/fun/awesome/cool/funny/amusing/exciting/enjoyable/fascinating and liked taking part. Children said ‘I never really liked science but now I do. I think it's really interesting’ and ‘When my teacher said “you are doing a science experiment” I got very excited’
Sensory engagement	The look and smell of the stimuli activities were important for children. Some liked it when it was messy or disgusting; others liked it when it was pretty, colourful or ‘looked magical’. Some children thought that the best CoSE classes were scientific and artistic. Children also liked going outside
Sharing	Children liked being able to do and discuss the stimuli themselves or at home and to share what they did with parents, for example, ‘my parents really enjoyed listening to what experiments we did’, and ‘you found out stuff and you could show your parents at home’. Children said that CoSE made them want to experiment more at school and at home, and that they would like it if science was a new subject in primary school. Children also liked working with others
Surprise	The surprise element to the CoSE stimuli was important for engaging children. They said that ‘I didn't know what would happen’, ‘I didn't know that could happen’ and ‘you would never think about doing that’. Children liked it when it was shocking/surprising/unexpected/weird. One child described CoSE as ‘fun things happened that were weird and exciting’
*Learning*	
Learning science	Children enjoyed learning science. Some children said it was better than other subjects ‘science is one of the most best things I have ever done in school’, and some said that it was ‘better than P4C’. Children said that they liked ‘learning and having fun’. Children said that ‘it helped me learn lots about Earth’ and ‘It was a fun way to learn about electricity. It benefitted me.’ and that they liked learning about things they have never heard of, and learning how things work. Some children saw a longer term benefit from their CoSE classes: ‘It has made me think about science more and hopefully will help me when I go to high school’
Thinking skills	For some children, the best part of CoSE was that it helped them develop skills, for example ‘It helped me learn how to communicate better with other people and to pick other people's ideas’; ‘I liked listening to other people's ideas’ and ‘I liked making up questions with other people.’ Some highlighted the connection between learning, engagement and working with others: ‘this was fantastic for us to get together and have fun even though we were still learning’
*Pedagogy*	
Practical	Most children highlighted doing or watching the practical/demonstration stimulus as the best part of CoSE. They reported that they liked thinking about what was going to happen, testing, seeing what happened and making observations. They also highlighted the atmosphere in the classroom: ‘it was best when the tension built’. Specific elements of practicals that children liked were when it was fast, dramatic or energetic, or involved fire, explosion or food, ‘when you got to talk about bubbles’ and ‘it was like dancing, and I like dancing’. Children also said that ‘these have made me love science’ and identified areas they would like to learn about through CoSE: ‘I'd love to do this about the environment.’ Some children highlighted the connection between the practical and their learning: ‘When we started some of the experiments I was bored but when I listened and participated I started to like it and enjoy it’
Group work	Children liked working in groups and as a whole class, as well as being able to work with their friends during the CoSE classes
Questions	For many children, it was the questions that they enjoyed. This included making up their own questions, thinking of questions in groups, choosing the question (e.g. using stickers, voting), and answering the question. They also liked it when their question was selected
Dialogue	Many children reported that the dialogue/discussion/talking was the best part of their CoSE classes. They liked it ‘when you got to the right answer without the teacher’, and said that ‘the discussion was best because I learnt more’. Children said that they ‘*loved* trying to figure out what happened’ and ‘find out how it works’ and being able to share and discuss their opinions. Many children thought that CoSE helped them to become more independent and challenged them: ‘It was a challenge to figure out what happened’ and ‘I liked that we did it ourself and the teacher doesn't help’
Active learning	Children liked doing different things during the CoSE lessons, particularly the starter games

**Table 2.** Worst aspects of CoSE for children at Key Stage 2.

*Engagement*	
Emotional engagement	Children did not like CoSE classes when they found it boring or not very exciting, cool, interesting or amusing
Sensory engagement	Sensory aspects of CoSE were highlighted by children in a negative light. Some children didn't like that it was messy, others didn't like the smell (of vinegar), touching balloons or holding hands with their peers
*Pedagogy*	
Practical/demonstration	Children did not like the CoSE classes when the stimulus didn't work (for example, when the raisins didn't dance) or when they had seen it before or knew what was going to happen. Others found it off-putting when the stimulus took a long time, when not much appeared to happen or when they experienced problems such as leakages. Some of the stimuli were described as too simple, not every scientific, too short, or too long, and others required ‘too many chemicals’. Some children said that ‘we didn't do it ourselves’
Dialogue	Children didn't like it when they didn't understand the enquiry, found it difficult or when they were asked to talk when they had no ideas. Some children particularly didn't like evaluating the dialogue
Writing	Although not a part of CoSE, a number of children said that they didn't like writing down what we saw and writing up after the experiments
Direct instruction	Children didn't like it when there was a lot of explanation from the teacher
Starter	Some children found the warm up games the worst part of the process and just wanted to get straight to the stimulus
Groupwork	Some children did not like that the teacher chose the groups for them to work in. One child said that the worst part of CoSE was ‘working with my partner because she never agreed with me’
Questions	Some children did not like generating questions, disagreeing on questions, or selecting a question. Where reasons were given this was ‘because there were so many things I wanted to know all of them’
*Organisation*	
Preparation	The worst aspect of CoSE for some children was setting up and rearranging the classroom, and waiting for something to happen
Frequency and duration of classes	Children said that the worst part of the CoSE was ‘the end because you just want more’. Others said that they were not doing CoSE often enough
*Respect*	
Class behaviour	The behaviour of the class was an important factor for children. They didn't like it when ‘people were standing’, when ‘people didn't listen’ or when ‘people did things before they were meant to’. They also objected to ‘people speaking when I was meant to be speaking’

Many children identified the best part of CoSE as relating to the practical, although some placed more value on the dialogue relating to the practical. Other important factors were related to children's emotional and sensory responses, the way that they were able to learn and the different ways of teaching during the CoSE lessons.

Children noted aspects of their CoSE that disengaged them: when class behaviour was poor, when the discussion was difficult, elements of groupwork, rearranging the room, and when there was a lot of teacher explanation and writing. This highlights the importance of the teacher's interpretation of and management of the CoSE.

Final evaluations completed by teachers validated the finding that children at Key Stage 2 were engaged by CoSE (Table 3). Of the 19 teachers returning questionnaires, all reported that the CoSE classes had engaged the children they teach. Fifteen said that the children enjoyed the classes, and 18 thought that the children found them interesting and helped them to talk about science. Teachers noted that the demonstration/practical stimulus in particular engaged the children, but also that the children enjoyed the freedom that they had to explore ideas and concepts without fear of failure. However, one teacher reported that children did not enjoy the discussion element of CoSE, and several noted that it took time for themselves and the children to get used to the discussion format.

**Table 3.** Teacher feedback on children's engagement with science through CoSE.

The children I teach …	SA (*n*)	A (*n*)	N (*n*)	D (*n*)	SD (*n*)
Engaged with science	8	11	0	0	0
Enjoyed CoSE classes	13	2	4	0	0
Were interested in CoSE classes	11	7	1	0	0
Talked about science	9	9	1	0	0

Note: Key: SA, strongly agree; A, agree; N, neither agree; nor disagree; D, disagree and SD, strongly disagree.

Comments made by teachers reinforced the view that CoSE engaged children:

The best thing was pupil engagement and ability to discuss and listen. It really challenged them to think.

I loved the experiments that caught the children's attention. The questions developed were also fantastic. They carried out extra individual research at home without prompting, cheered when they were told we were doing another session and asked fantastic questions. I loved this and so did the children.

The class in general have become more motivated by the enquiry lessons. They are keen to participate. It has encouraged pupils to ask more challenging questions and find out more. The investigations get the class buzzing. They talked readily about what they had seen and generated questions. The children were enthused and they carried out extra individual research at home without prompting.

The majority of teachers (14 out of 17) reported changes to the class and individuals within the class in relation to their engagement. They noted that the CoSE classes motivated the children; that ‘a real buzz about science was created’. They said that the class were keen to participate and that they were encouraged to ask more challenging questions and to want to find out more. ‘They can see science in the real world more.’ Teachers said that a wider range of children became engaged, and that there was more dialogue between children as a result of CoSE. Teachers also reported that children had more confidence to take risks, speak out, express their opinion and take part in discussions, particularly the academically less-able children. Children who would normally remain quiet had contributed opinions. These findings correspond with large-scale studies on engagment that have highlighted an association between teaching and learning activities with engagment in science (Hampden-Thompson and Bennett 2013), and particularly that engagement improves where teaching is less didactic and incorporates student voice (Lyons 2006) and collaboration with peers (DeWitt and Osborne 2008).

A CoSE based on the exploration of children's questions relating to the observation of a scientific phenomenon appears to be a successful strategy for promoting engagement with science at Key Stage 2. Teacher preparation in terms of making sure the demonstrations work (or being prepared to base the enquiry on the demonstration not working) is key.

### Learning through CoSE

To limit the length of the questionnaire for primary children, a ‘snapshot’ was taken rather than construct scales of learning in science and learning to question.

Most children agreed or strongly agreed that CoSE helped them think more about science (80%) and that because of their classes, they knew more about science (81%). They also reported that CoSE helped them to ask questions that can be explored (77%), to think about how questions can be answered (72%) and to give better reasons for their opinions (60%). They were less positive about the impact of CoSE on their ability to give better reasons than the other indications of learning. This is a skill that takes time to develop:

I found it a better way to learn.

The scientific enquiry classes have helped me to learn more about science.

I have really enjoyed learning a lot about scientific enquiry. It has really made me think a lot. It was wonderful doing them with my friends so we can discuss them together.

Teacher feedback validated the finding that CoSE helped children learn about science. All 19 teachers said that the children they teach had made progress in their understanding of science topics, ability to justify their own points of view and to question other points of view (Table 4).

**Table 4.** Teacher feedback on children's learning through CoSE.

The children I teach made progress in their …	L (*n*)	S (*n*)	NM (*n*)	N (*n*)
Understanding of science	6	13	0	0
Ability to justify their own point of view	7	12	0	0
Ability to question other points of view	5	14	0	0

Note: Key: L, lots; A, some; NM, not much and N, none.

Teachers' comments supported the claim that children made progress in their learning through CoSE. They reported that this was due to the dialogic enquiry which enabled them to share knowledge, learn from asking questions and making mistakes and inspired them to conduct independent research:

Children learnt new facts and through discussion and debate they connected experience to newly acquired knowledge. [CoSE] enhanced children's ability to learn from their mistakes.

Children are more enquiring and have developed questioning skills. They are now more ready to use scientific language to explain what they have seen. They go off and research on their own if they feel we have not explained the reasoning.

My children have been able to talk about some scientific concepts which would never have arisen through their WAU topics. Scientific understanding is growing with every CoSE.

In terms of learning, the majority of teachers identified changes to their class (*n* = 17) and to individuals (*n* = 14) likely to be attributable to CoSE. Teachers reported that children had developed their scientific questioning, vocabulary and understanding as well as their ability to make observations. One teacher noted that a child ‘with poor literacy skills demonstrated great oracy and understanding’ during the CoSE classes. Another reported:

CoSE has developed children's scientific knowledge very well. Children are retaining knowledge much better and can relay it orally several weeks after doing enquiry sessions. They have brought in research on Richard Branson and the Montgolfier brothers off their own back.

This supports evidence from research studies that have found that the development of enquiry skills helps to develop children's understanding of core scientific concepts (Skamp 2012), although further studies are required to sustain this argument in relation to CoSE.

Likewise, teachers noted the development of a number of key thinking skills identified in the revised curriculum for Key Stage 2, including problem solving, questioning, justifying opinions and critical thinking. One teacher noted that ‘children have developed a much more forensic attitude towards all questions and problem solving’, and another said that ‘children really blossomed in terms of their thinking skills and ability to formulate and argue opinions’. In common with other studies, this presents some evidence to suggest that a focus on thinking and learning can enhance subject content (Department for Education and Skills 2005) and that the use of dialogue of this nature in science can improve children's scientific reasoning (Sprod 1998).

### The impact of CoSE on teaching in Key Stage 2 classrooms

In addition to the impact CoSE had on children, teachers reported that participating in the study enhanced their ability to teach science: all said that they found CoSE useful and would continue to use it. They noted becoming more relaxed knowing that they were not trying to force-feed facts, and said that CoSE allowed them to address more difficult concepts in a child-friendly way, and that it gave them the confidence to allow children more control over their learning. They also noted that they were able to determine what children knew and give them the opportunity to find things out for themselves. Teachers said that they had become aware that children are afraid to make mistakes and the project had helped them and the children to overcome this. Through their use of the CoSE strategy, teachers found that their children became more open minded and better thinkers. A common idea expressed by teachers is that:

My pupils are excited by the notion of doing a CoSE lesson. They love the fact that it is practical with loads of thinking … many pupils have become increasingly confident in sharing their opinions. CoSE has encouraged my pupils to reason out why they think the way they do – it has pushed them to come up with a scientific theory, even if it might not be correct.

Teachers also identified several limitations to the use of the CoSE strategy. Some found it difficult to find appropriate space or fit CoSE into curriclum time. Others found it a challenge to establish the ground rules for discussion and to encourage children to create and select good questions. Others thought that the restriction of the discussion to one question was a limitation. When taught as isolated lessons, one teacher felt that this might give the children misconceptions. The main limitation highlighted by teachers was that they had only been provided with eight lesson ideas: they wanted more topics and more resources, connected to their own WAU themes.

## Conclusions

Evaluation of the CoSE strategy revealed that children found it engaging. They enjoyed the classes, found them interesting, and carried on their discussions and further investigative work beyond the classroom. They were keen to share what they had learnt with their parents. Children also thought that CoSE helped them to learn more about science and to work with other children.

Teachers found the CoSE strategy useful for engaging children, supporting learning and developing children's social skills. However, limitations identified by teachers indicate the need for more training in how to facilitate discussions, including how to deal with misconceptions and how to make the dialogue more probing.
